# Alcohol and the Developing Brain: Why Neurons Die and How Survivors Change

**DOI:** 10.3390/ijms19102992

**Published:** 2018-09-30

**Authors:** Alberto Granato, Benjamin Dering

**Affiliations:** 1 Department of Psychology, Catholic University, Largo A. Gemelli 1, 20123 Milan, Italy; 2 Faculty of Natural Sciences, University of Stirling, Stirling FK9 4LA, UK; b.r.dering@stir.ac.uk

**Keywords:** fetal alcohol, GABA, ethanol, cerebral cortex, pyramidal neurons, apoptosis

## Abstract

The consequences of alcohol drinking during pregnancy are dramatic and usually referred to as fetal alcohol spectrum disorders (FASD). This condition is one of the main causes of intellectual disability in Western countries. The immature fetal brain exposed to ethanol undergoes massive neuron death. However, the same mechanisms leading to cell death can also be responsible for changes of developmental plasticity. As a consequence of such a maladaptive plasticity, the functional damage to central nervous system structures is amplified and leads to permanent sequelae. Here we review the literature dealing with experimental FASD, focusing on the alterations of the cerebral cortex. We propose that the reciprocal interaction between cell death and maladaptive plasticity represents the main pathogenetic mechanism of the alcohol-induced damage to the developing brain.

Early exposure to alcohol during pregnancy, whose effects are usually referred to as fetal alcohol spectrum disorders (FASD), is highly disrupting for the development of the nervous system and is among the main causes of intellectual disability in Western countries [1,2].

The role of excessive neuron death in generating the damage that characterizes FASD was established by the first experimental works based on early alcohol exposure (e.g., [3,4]). Afterwards, a seminal paper from John Olney′s lab [5] described widespread apoptosis after late prenatal or neonatal exposure and attributed this phenomenon to the action of alcohol on the two chief neurotransmitter systems of the brain. Alcohol, in fact, is able to induce both the block of NMDA receptors [6,7,8] and the positive modulation of GABA transmission (see [9], for review). This dual mechanism is also shared by other anesthetic drugs acting on the central nervous system [10]. In the following years, the excessive inhibition hypothesis was repeatedly challenged and it was suggested that alternative mechanisms can be operant in some cell populations [11,12,13]. The cell death is not necessarily and exclusively caused by impairments of the neurotransmitter system. Indirect effects, such as those related to the disruption of brain or uterine vasculature, may play a role in alcohol-induced cell death [14,15,16].

Furthermore, the neuron depletion itself is not the only factor leading to the devastating effects of alcohol on the developing brain. When a human community loses many of its citizens (e.g., from the effects of war), the deleterious effects on the society are due not only to the direct consequences of the casualties, but also to the fact that the survivors may react in a “maladaptive” way (either from the psychological or the social point of view). By analogy, this mini-review/commentary is focused on the assumption that, even though the direct effects of neuron death following in utero exposure to alcohol can be dramatic per se, some of the long-term consequences observed in experimental or human FASD are due to an anomalous (or also maladaptive) plastic rearrangement of surviving neurons. Within such a frame, neuroapoptosis and reorganization of brain structure and function can interact in a vicious circle, sustaining and amplifying each other.

## 1. Brain Plasticity, Friend or Foe?

There are several types of brain plasticity. The prototypical one, and among the most important, was discovered during the 70s of the last century by Bliss and Lomo, is referred to as long term potentiation (LTP) and can be described as a long lasting increase of the synaptic efficacy after tetanic stimulation [17]. Since then, several other forms of plasticity have been described. They include a refinement of the concept of synaptic plasticity, based on the synapse potentiation or depression according to the relative timing of pre- and postsynaptic spike (spike timing dependent plasticity—STDP [18,19]). Another kind of neural adaptation, called intrinsic plasticity, is represented by the change in intrinsic excitability of neurons and, as such, it is linked to modulation of voltage-gated rather than ligand-gated synaptic channels [20]. Finally, the plastic modifications can be also represented by widespread rewiring of connections that usually occur during critical periods of brain development [21]. The prevailing view in neuroscience is that most neurological and mental diseases can be reinterpreted as conditions in which plastic changes to the brain are anomalous, not necessarily defective (see Figure 1 in [22]; see also [23]). The consideration that brain plasticity might not be solely reparative in nature represents a challenge to the Kennard principle—the seminal papers by Margareth Kennard, published in the first half of the 20th century, state that a vigorous remodeling of brain functions and connections, such as that occurring after early injury, makes the brain more resilient to the induced damage [24]. In the following years, this principle has been matter of debate and often criticized [25,26].

## 2. Early Alcohol Exposure and Cell Death

The apoptosis that follows early exposure to ethanol is widespread [5], but is far from affecting all the cell populations to the same degree. One of the most damaged structures is the cerebral cortex, where different protocols of alcohol exposure carried out in different experimental animals lead to converging results: pyramidal neurons of layer 5, the main source of output from the cortex, are more susceptible to the death-inducing effects of alcohol, as compared to principal neurons that reside in other cortical layers [27,28]. As to the GABAergic cortical interneurons, although being a minority, they represent a more heterogeneous population, when compared to the pyramidal neurons. Accordingly, results concerning FASD and cortical interneuron death are more controversial and are likely to be highly dependent on different protocols and timing of experimental alcohol exposure. While no change in the number of parvalbumin immunoreactive interneurons was found after alcohol exposure spanning P2–P6 in rats [29], Smiley and coworkers observed a substantial reduction of the same interneuron population after P7 exposure in mice [30]. What makes the picture of inhibitory interneurons even more puzzling, is that, possibly due to a lack of naturally occurring cell death, some interneuron populations are increased after alcohol exposure. This is the case for parvalbumin neurons in the prefrontal cortex after prenatal exposure in mice [31], as well as for calretinin interneurons in the sensori-motor cortex after postnatal exposure in rats [29]. It is thus likely that, besides inducing cell death, ethanol can, in some instances, delay maturation processes, keeping some populations from undergoing a physiological reduction of elements. This might be the case for the calretinin subset, whose normal fate is to decline during normal postnatal development [32]. An alternative hypothesis is represented by an alcohol-induced change of migration speed, that might result in an interneuron population to be more represented in some brain regions and less in others [33] (see also below).

There might be long-lasting consequences of the differential effects of alcohol on the survival of specific cell populations. For instance, besides the acute potentiation of GABA transmission elicited by alcohol exposure, the increase of parvalbumin interneurons leads to a permanent shifting of the excitatory/inhibitory balance toward inhibition [31].

## 3. Relationship between Cell Death and Plasticity

Some of the molecules that mediate cell death are also deeply involved in neural plasticity. Therefore, the same molecular network responsible for alcohol-induced apoptosis can also modify the neural plasticity and, consequently, the wiring of the brain. The p75 low-affinity neurotrophin receptor (p75-NTR) is overexpressed in the sensori-motor cortex of adult rats exposed to ethanol during the first postnatal week [28]. The p75-NTR is also highly increased in human neuroblastoma cells treated with ethanol and the use of interference RNA targeting p75-NTR reverses the proapoptotic effect of ethanol [34]. A similar protective effect of p75 inhibition has been demonstrated in primary cultures of neurons exposed to the proapoptotic effect of the anesthetic isoflurane [35]. The neuron death-inducing role of p75-NTR has also been demonstrated for other conditions, such as intracerebral hemorrhage [36] and oxidative stress [37]. At the same time, the low-affinity neurotrophin receptor is also a key molecule of plastic remodeling. In fact, the p75-NTR signaling pathway has been shown to modulate synaptic plasticity and consolidation in organotypic cultures of the mouse hippocampus [38]. Woo et al. reported that activation of p75-NTR enhances hippocampal long term depression (LTD) [39]. In addition, both the dendritic architecture and neurite elongation are modulated by p75 signaling [40,41].

Other molecules that mediate apoptosis, such as caspase-3, have been reported either to be upregulated [42,43,44,45,46] or to display changes of their developmental timing profile after exposure to ethanol [47]. Caspase 3, besides being involved in neuron death, turns out to be also involved in spine remodeling and other types of plasticity [48,49,50].

Some of the experimental works cited above point out that both prenatal [47] and early postnatal exposure to alcohol [28] can be responsible for the activation of apoptosis-related molecules lasting well beyond the period of exposure, up to adulthood. The fact that even a narrow and early exposure window can result in a long-lasting, possibly permanent, susceptibility to neuron death, circuit anomalies, and plastic changes, is one of the most intriguing issues raised by the neurobiological studies on FASD and will be addressed in the next paragraph.

The increase of p75-NTR and caspase 3 signaling are not the only candidates to link apoptosis and developmental plasticity. Early alcohol exposure has been shown to induce changes of the neurotrophin signaling system [51]. Either a decrease [52,53] or an increase [54] of BDNF neurotrophic support has been demonstrated after prenatal or postnatal exposure to ethanol, respectively. The BDNF-TrkB signaling is obviously involved in apoptosis [55] and its direct involvement in FASD-related changes of hippocampal plasticity has been shown by Zucca and Valenzuela [56]. In addition, the well-established role of BDNF-TrkB in dendritogenesis [57,58] can account for the alterations of dendritic branching observed in experimental FASD [59,60].

## 4. A Vicious Circle to Maintain Long-Lasting Effects of Ethanol

As we have pointed out in the previous paragraph, early exposure to alcohol can activate molecular pathways such as the p75-NTR signaling which in turn, besides triggering cell death, can also modify synaptic plasticity. Both LTP and LTD, the best known instances of synaptic plasticity, are able to affect the brain wiring, through the stabilization, neoformation, or elimination of dendritic spines [61,62,63]. Actually, there are reports of impaired LTP and LTD after early exposure to ethanol [64,65,66]. As a consequence, a reduction of spine density represents one of the major anatomical features observed in FASD [67,68]. Other factors elicited by alcohol exposure during development can modify the response of the brain to developmental cues and its wiring. As already pointed out in a previous paragraph, the relative proportion of the different populations of GABAergic interneurons can be altered during FASD [29,31]. GABA neurotransmission is known to play a pivotal role in the modulation of developmental plasticity [21,69,70]. Therefore, the impairment of GABAergic transmission might be responsible for microcircuit alterations observed in experimental FASD, such as the disruption of ocular dominance plasticity in the visual cortex [71,72].

The change of network activity due to the modification of excitation/inhibition balance [31] can affect the trafficking and phosphorylation of ion channels, that have been shown to be activity-dependent [73,74,75]. The dendritic excitability at the calcium electrogenesis zone of the apical dendrite is in fact permanently impaired after early postnatal exposure to alcohol [76] and the reduction of dendritic excitability and calcium spikes can lead to a further impairment of network activity and synaptic plasticity [77]. Moreover, voltage-gated calcium channels, responsible for the generation of calcium spikes, have been also shown to modulate dendritic spine morphology [78]. Other ion channels, including those gated by neurotransmitters, display long-lasting changes after early exposure to ethanol [79,80].

**Figure 1:**
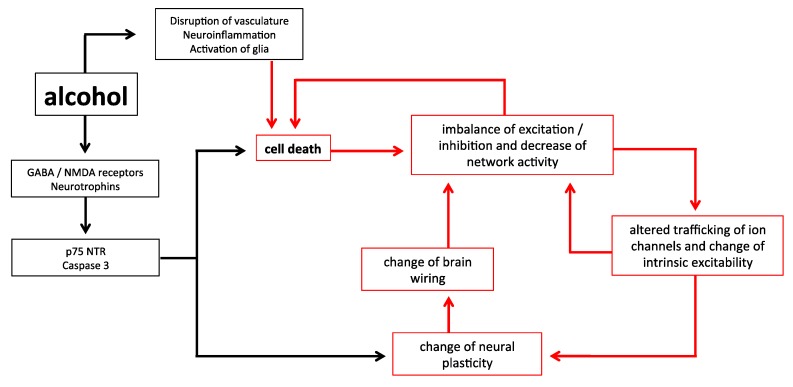
A schematic of the relationships between early alcohol exposure and cell death. Alcohol has immediate impacts upon the system which can lead to an increased apoptosis different from natural programmed cell death, yet also results in plastic changes to the brain. This can lead to a further imbalance of the system in terms of inhibition/excitation and cell excitability, creating a vicious cycle likely to result in further abnormal apoptosis. Critically, the impact of alcohol on all these factors is mediated by the duration of exposure, and the time of exposure in the developmental trajectory.

The relationship between early alcohol exposure and neural activity is complex. In immature neurons, due to the relatively high concentration of intracellular chloride, GABA is excitatory [81]. Therefore, in line with the known effect of GABA positive modulation described in adults, the activity of immature neural networks can be increased as a consequence of alcohol exposure [82]. However, it should be considered that the early effect of GABA in vivo and in slice preparations might be different. In fact, the GABAergic transmission in vivo is already inhibitory during the first postnatal week [83]. Conversely, the depressant effect of ethanol on NMDA currents has been demonstrated also at early stages of development, remains after alcohol withdrawal [79], and can contribute to depressing neural activity [84]. At later stages of maturation, when GABA is inhibitory, the effect of interneuronopathy [29,31], combined with the FASD-related alteration of glutamatergic cortico-cortical connections [60] can further depress the network activity. This reduction of activity, in turn, represents a strong trigger of apoptosis [85,86,87,88,89]. This mechanism can explain the long-lasting susceptibility of cortical neurons to apoptosis, even when they are no longer exposed to ethanol. An increased rate of apoptosis might, in turn, further decrease neural activity. This vicious circle (illustrated in Figure 1), elicited at very early stages, can maintain apoptotic phenomena at later stages of development. It is not clear whether this mechanism continues to be operant in more mature, adult brains. However, a depressive effect on the electrical activity of pyramidal neurons after acute exposure to alcohol has been observed in the adolescent/adult brain [90,91] and is also accompanied by an increased rate of apoptosis, similarly to what happens for early exposure [92,93,94]. Layer 5 pyramidal neurons of adult rodents, which are strongly impaired in FASD experimental models [60,76], undergo extensive death also in some animal models reproducing Alzheimer′s disease, with cell degeneration preceded by a reduction in electrical activity of these neurons [95]. Furthermore, in models of Parkinson′s disease based on the administration of the toxin 6-hydroxydopamine, Huang et al. [96] observed hypoexcitability of dopaminergic neurons and increase of apoptosis. Similar mechanisms, related to endoplasmic reticulum stress and calcium dishomeostasis, have also been hypothesized for ethanol toxicity [97,98]. However, it is not clear whether, in mature brains, hypoactivity and apoptosis are just simultaneous events or are reciprocally affected and amplified.

Epigenetic mechanisms represent good candidates to explain the long-lasting effects of ethanol in promoting apoptosis (reviewed in ref. [99]). Subbanna et al. [100,101] demonstrated that exposure to ethanol during early postnatal life is able to induce apoptosis through histone acetylation and dimethylation. Most of the epigenetic effects induced by ethanol last for a long time after alcohol withdrawal [102,103]. In addition, neuron activity is also able to affect the epigenome, for instance through action on the nuclear/cytoplasmic shuttling of the histone deacetylase HDAC4 [104,105], whose accumulation in the nucleus can cause apoptosis [106].

## 5. Alcohol May Enhance the Response of Glial Cells

Although often underestimated, glial cell alterations play a key role in the genesis of FASD (see [107], for review). There is a growing body of work assessing glial cell responses during early ethanol exposure (pre- and postnatal). The loss of neurons through apoptosis is also compounded by the response of microglia, which may lead to further cell death. Briefly, microglia themselves are resistant to the damaging effects of ethanol, and their function of removing dead cells is not affected by the presence of ethanol in acute exposures; morphological effects upon microglia themselves appear to be transient in nature [108]. Ahlers et al. [108] also observed transient increases in pro-inflammatory factors (PIF) TNFα and IL-1β after heavy alcohol exposure but concluded that this was not directly related to the presence of alcohol, rather, due to increased neuroapoptosis. These findings lead to the view that the microglial cells do not contribute directly to neurodegeneration, rather they serve a protective function after alcohol damage to the cortex [109]. However, contrasting work by Fernandez-Lizarbe et al. [110] shows that ethanol upregulates expression of toll-like receptors 2 and 4 (TLR2, TLR4) in glial cells, which normally serve to activate immune response through the binding of microbes/pathogens. In the case of acute alcohol exposure, the activation of TLR 2 and 4 leads to the release of inflammatory cytokines, the result of which is an increased neuro-inflammatory response in the presence of alcohol. Chronic microglial activation may lead to further cell death, since over time the release of pro-inflammatory cytokines can be toxic [111]. Glial cell activation may also show regional differences in the presence of alcohol, for example, with accelerated degradation of cerebellar Purkinje cells compared to hippocampal neurons [112], yet this may be more representative of the time of exposure to alcohol during particular stages of development, i.e., animal models of FASD usually test rodents in an age range from P3–P11, equivalent to the third trimester in humans. Further evidence from Cantacorps et al. [113] suggests that motor coordination difficulties and spatial memory deficits are observable in pre-natal alcohol exposed mice, who were also weaned by mothers who continued to drink alcohol. These behavioral deficits corresponded with increased neuro-inflammation (expression of TLR2 and 4) in the hippocampus (also showed an increase in expression of IL-1β) and prefrontal cortices. A significant increase in activated caspase-3 was also observed, suggesting alcohol-induced apoptosis [113]. In summary, while activation of glial cells normally serves to protect neural functioning, observed in single acute exposures to alcohol, there is evidence that alcohol may enhance neuro-inflammation and under conditions of prolonged activation, for example, during chronic alcohol abuse, could possibly lead to further cell death due to the toxic effects of PIFs.

## 6. Alcohol Affects the Developmental Trajectory of Cortical Neurons

It should be pointed out that not all the mechanisms leading to alcohol teratogenic effects are necessarily related to apoptosis. Heterotopias of cortical neurons, presumably due to defects of cortical migration, have been observed in the first studies of experimental FASD [114]. The migration of glutamatergic neurons to the proper cortical layers occurs radially from the ventricular zone of the pallium, with cells first resting in deep layers, and lastly in layer 2/3, often referred to as “inside-first, outside-last” gradient. In contrast, GABAergic interneurons are chiefly generated in the medial and caudal ganglionic eminence, and migrate tangentially through cortical layers (for a review see [115]). The medial ganglionic eminence is the main source of parvalbumin interneurons, basket and chandelier cells, whereas calretinin or vasoactive intestinal peptide (VIP) interneurons such as bipolar or double bouquet cells are generated in the caudal ganglionic eminence [116]. Migration of cortical neurons is driven by the presence of both GABA and glutamate in the environment acting as chemical attractants [117,118]. It is thus likely that alcohol affects the embryonic migration of cortical neurons through its known action on GABA and NMDA receptors. Besides the effect on the migration of interneurons, observed by Cuzon et al. [33], alcohol-induced disruption of the radial migration of pyramidal neurons has been also described [119].

Another aspect of alcohol-induced damage is represented by the alteration of cell cycle kinetics and cell proliferation occurring during prenatal life [120,121]. It has also been suggested that apoptosis and cell cycle alteration can coexist in the embryonic brain, but are mediated by separate mechanisms [122].

It is worth mentioning here that the timing of developmental exposure to alcohol is a critical factor in determining whether apoptosis represents the main pathogenetic factor of FASD. In fact, while early postnatal exposure during synaptogenesis has been demonstrated to trigger extensive apoptosis [5], immature neural precursors are relatively resistant to ethanol-induced apoptosis [123].

## 7. Conclusions

The evidence highlights that different experimental protocols affect both the pathogenetic mechanisms and the outcome of FASD, the timing of exposure being among the main determinants of such a difference. Evidently the Kennard principle does not apply in FASD, where early exposure to alcohol is more damaging to the development of the neocortex, for example through enhanced apoptosis, toxic neuro-inflammation, altered neural migration, and cell cycle kinetics. Not only is the rate of apoptosis influenced by the exposure period, but also the circuit plasticity seems to be dramatically different according to whether alcohol is given in rodents prenatally or postnatally. For instance, a binge exposure during prenatal life in mice results in a transient decrease of dendritic complexity in pyramidal neurons [119], whereas a permanent reduction of basal dendritic branching is observed in the same neurons after early sustained postnatal exposure [60]. Once again, the interplay between cell death and modified developmental plasticity is clearly a key feature of FASD, and a better knowledge of this interaction is needed which incorporates evidence from acute/binge or heavy sustained drinking paradigms. Keeping women from drinking any alcohol during pregnancy should be the first effort of public health policy, but this is not easy. Therefore, basic research on experimental models of FASD can provide clues for developing new therapeutic strategies to reduce direct and indirect consequences of alcohol-induced cell death.
